# Whole genome sequencing data of native isolates of *Bacillus* and *Trichoderma* having potential biocontrol and plant growth promotion activities in rice

**DOI:** 10.1016/j.dib.2022.107923

**Published:** 2022-02-04

**Authors:** C. Kannan, M. Divya, G. Rekha, Kalyani M. Barbadikar, P. Maruthi, S.K. Hajira, R.M. Sundaram

**Affiliations:** ICAR-Indian Institute of Rice Research, Rajendranagar, Hyderabad 500030, India

**Keywords:** Biocontrol agents, *Bacillus*, *Trichoderma*, Whole genome sequencing

## Abstract

Six native isolates of *Trichoderma* and *Bacillus* having potential for biocontrol and plant growth-promoting activities in rice were isolated from different rice growing regions of India. These isolates were screened for their efficiency in both *in vitro* and *in vivo* conditions for three years. The identity of the isolates was confirmed both by morphological and molecular characterization. Three *Bacillus* spp. *viz., Bacillus velenzensis* strain BIK2, *Bacillus cabrialesii* strain BIK3 and *Bacillus paralicheniformis* strain BIK4 and *Trichoderma* spp. *viz., Trichoderma asperellum* strain TAIK1, and *T. asperellum* strain TAIK5, native to the Telangana state, in Southern India except for strain TAIK4 (Rewa district in the state of Madhya Pradesh in Central India). These promising isolates were subjected for whole genome sequencing using the Illumina platform and data was presented. The data was emanated for *Trichoderma asperellum* (TAIK1), *Trichoderma asperellum* (TAIK4), *Trichoderma asperellum* (TAIK5), *Bacillus velezensis* (BIK2), *Bacillus cabrialesii* (BIK3) and *Bacillus paralicheniformis* (BIK4) isolates had an average 100X coverage of 109X, 150X and 116X; 1447X, 905X and 585X respectively. Further studies on the annotation of the data obtained in correlation with the lab and field performance of these microbes would enable them to be used in metagenomics studies to compare their performance under natural conditions with different microbiota and popular rice varieties. Bioformulation of these strains would be more appropriate with the availability of this genomic data.

## Specifications Table

**Table:** 

Subject	Microbiology
Specific subject area	Biocontrol agents, antagonists
Type of data	Assembly (fasta files), Tables, Figures
How data were acquired	Whole genome sequencing conducted on Illumina HiSeq 2500 instrument platform
Data format	Raw data
Parameters of data collection	The microbes were isolated from the rhizosphere soil of rice in the farmers’ fields. Genomic DNA was extracted from pure culture of individual isolates
Description of data collection	Total genomic DNA was isolated from three *Bacillus* and three *Trichoderma* spp., purified and subjected to HiSeq Illumina sequencing (2*150 bp) *de novo* assembly.
Data source location	*Trichoderma asperellum* (TAIK1)- Hyderabad *Trichoderma asperellum* (TAIK4)- Rewa *Trichoderma asperellum* (TAIK5)- Hyderabad *Bacillus velezensis* (BIK2)- Karimnagar *Bacillus cabrialesii* (BIK3)- Hyderabad *Bacillus paralicheniformis* (BIK4)- Nalgonda
Data accessibility	Data is publicly available at NCBI GenBank from the following links: Assembly accessions and Bio project accessions https://www.ncbi.nlm.nih.gov/assembly/GCF_019336145.1/ https://www.ncbi.nlm.nih.gov/assembly/GCF_018829645.1/ https://www.ncbi.nlm.nih.gov/assembly/GCF_019336205.1 https://www.ncbi.nlm.nih.gov/assembly/GCA_019594945.1 https://www.ncbi.nlm.nih.gov/assembly/GCA_019594925.1/ https://www.ncbi.nlm.nih.gov/assembly/GCA_019481625.1/ Bio project IDs PRJNA744701- BIK2- *Bacillus velenzensis* PRJNA735062- BIK3- *Bacillus cabrialesii* PRJNA744714- BIK4- *Bacillus paralicheniformis* PRJNA727916- TAIK1-*Trichoderma asperellum* PRJNA735060- TAIK4- *Trichoderma asperellum* PRJNA745529- TAIK5- *Trichoderma asperellum*
Related research article	C. Kannan, M. Divya, G. Rekha, P. Maruthi, Hajira Shaik and R. M. Sundaram, Diversity analysis of antagonistic microbes against bacterial leaf and fungal sheath blight diseases of rice. Egypt J Biol Pest Control. 31(2021) 115. doi:10.1186/s41938-021-00462-x

## Value of the Data

• This whole genome sequence data of six isolates of native biocontrol agents *viz.*, three *Bacillus* and three *Trichoderma* isolates serve as an important source towards an understanding of these bioagents which suppress the plant pathogens like *Rhizoctonia solani* and *Xanthomonas oryzae* pv. *oryzae* in rice and in addition induces plant growth promotion in rice.
• The data is useful in the annotation of the genes involved in the pathways of enzymes, effector proteins and metabolites/alkaloids, involved in the bioagent-host plant-pathogen interactions from the perspective of these antagonistic bioagents
• The data provides valuable information on these native bioagents and enables their efficient use by all the stakeholders including the biopesticide industries to use them as biocontrol agents and as biofertilizers in sustainable eco-friendly cultivation of rice. The genomic data of these potential bioagents submitted will help in the breeding of cultivars that respond well to the bioagents when applied. For instance, TAIK1 application on 30^th^ day of transplantation released growth promoting substances and also suppress the infection induced by *R. solani* and *S. oryzae.* It has also been reported that the bioagents application needs to be standardised for different varieties [1].

## 1 Data Description

Biological control is the process of using friendly bioagents or their products to suppress the pathogens leading to the sustainable integrated management of plant diseases [2]. Species belonging to the genera *Trichoderma, Bacillus* and *Pseudomonas* are more commonly found in the plant rhizosphere that helps in the growth promotion of the plants and induces resistance/tolerance against biotic and abiotic stresses. Members of the genus *Bacillus*, a common soil saprophytic gram-positive bacterium and *Trichoderma* a saprophytic fungus in rhizosphere soil, are used for their plant growth promotion and biocontrol qualities that make them a better alternative to chemical pesticides in long term use [3].

**Fig. 1:**
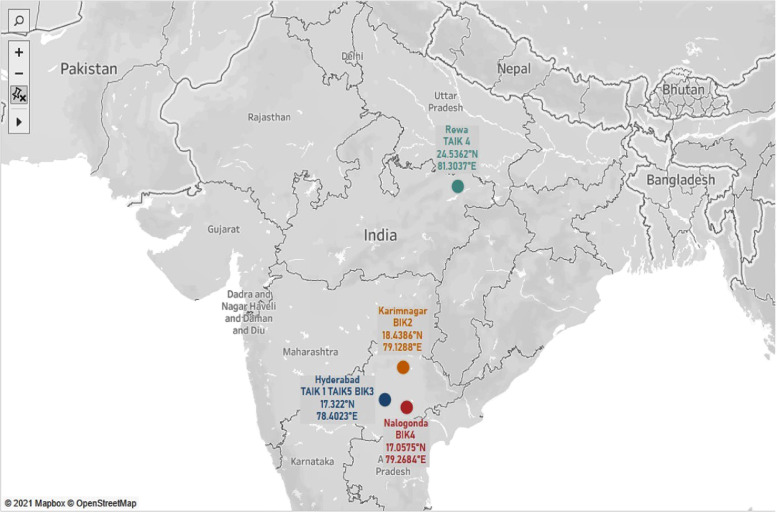
Illustration of map indicating the location of the strains collected from India (Tableau public 2021.2).

In this manuscript, we report the whole genome sequencing (WGS) data of three *Bacillus* isolates (BIK2, BIK3 and BIK4) and *Trichoderma* isolates (TAIK1, TAIK4 and TAIK5) collected from different states of India using standard dilution method [4]. The geographic data of the sampling sites and the origin of the isolates are represented as Fig. 1. Detailed statistics of three *Bacillus* isolates *viz.,* BIK2, BIK3 and BIK4 and three *Trichoderma* isolates *viz.,* TAIK1, TAIK4 and TAIK5 were presented in Tables 2 and 3.

## 2 Experimental Design, Materials and Methods

### 2.1 Culture and DNA extraction

**Fig. 2:**
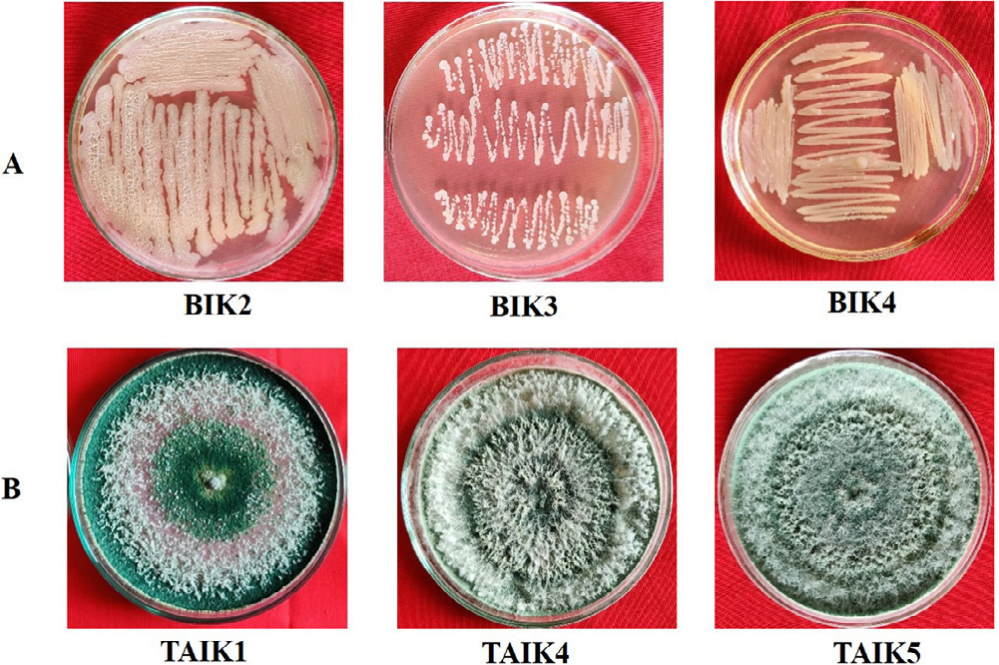
Culture plates of (A) *Bacillus isolates* (B) *Trichoderma isolates*.

*Bacillus* and *Trichoderma* isolates were obtained from the rice rhizosphere of different regions of India, using the standard serial dilution method (Fig. 1). *Trichoderma* specific medium (TSM) and peptone yeast extract medium (PYEM) was used as a selective medium for the isolation and purification of fungal and bacterial antagonists, respectively [4]. Key morphological and microscopic characters were used for the identification of *Trichoderma* isolates [5] and *Bacillus* isolates [6] (Fig. 2; Table 1). For whole genome sequencing, genomic DNA from the three *Bacillus* and three *Trichoderma* strains were isolated using DNA isolation kit NucleoSpin® microbial DNA kit as per the manufacturer's protocol (Macherey-Nagel, Germany). The DNA libraries for Whole Genome Sequencing were processed using standard protocols and sequenced using the HiSeq 2500 instrumentation platform (Agri Genome Labs Private Limited, Kochi, India).

**Table 1:** Morphological identification of *Bacillus* and *Trichoderma* isolates.

		Colony morphology			Sporulation	
Isolate code	Scientific name	colour	Area covered by radial growth of colonies in 36 h (mm)	Texture	Colour of spores	Days for maturation
BIK2	*B. velezensis*	Grey white	15.0 ± 0.03	Round, smooth and moist	-	-
BIK3	*B. cabriesii*	Of-white	21.0 ± 0.10	Flat, opaque and dry	-	-
BIK4	*B. paralicheniformis*	Pinkish whit	18.0 ± 0.09	Irregular and extra slimy	-	-
TAIK 1	*Trichoderma asperellum*	Dark green	37.0 ± 0.12	Smooth mat with concentric rings	Yellowish Green	4
TAIK 4	*Trichoderma asperellum*	Dark green	41.0 ± 0.10	Fluffy mat	Dark green	3
TAIK 5	*Trichoderma asperellum*	Dark green	45.0 ± 0.04	Smooth mat	Dark green	2

The table is modified from Tables 1 and 2 from the article referred - 10.1186/s41938-021-00462-x[1].

**Table 2:** Assembly Statistics of three *Bacillus* and *Trichoderma* isolates.

Attributes/ Statistics	*Bacillus velenzensis*	*Bacillus cabrialesii*	*Bacillus paralicheniformis*	*Trichoderma asperellum*	*Trichoderma asperellum*	*Trichoderma asperellum*
Isolate	BIK2	BIK3	BIK4	TAIK1	TAIK4	TAIK5
Contigs	26	28	30	702	473	449
Largest contig	10,78,503	5,75,880	10,56,155	10,48,585	6,24,435	7,25,734
Total Length	39,00,416	41,08,741	44,18,047	3,72,93,549	3,99,77,543	3,60,36,647
N50	10,29,777	3,20,958	6,27,466	2,26,906	2,07,650	1,61,701
N75	4,40,514	1,91,033	2,26,402	1,14,355	1,07,158	87,099
L50	2	5	3	50	64	70
L75	4	10	6	109	132	144
GC%	46.52	44.08	45.47	47	48	49

**Table 3:** Genome features of three *Bacillus* and *Trichoderma* isolates.

Genome features and gene ontology	*Bacillus velenzensis*	*Bacillus cabrialesii*	*Bacillus paralicheniformis*	*Trichoderma asperellum*	*Trichoderma asperellum*	*Trichoderma asperellum*
Isolate	**BIK2**	**BIK3**	**BIK4**	**TAIK1**	**TAIK4**	**TAIK5**
Protein coding genes	3751	4095	4495	11,592	14,174	11,589
Biological processes	1077	2095	2074	4051	5686	4045
Molecular functions	2111	4120	4228	10,717	14,080	10,692
Cellular components	863	1837	1801	4480	5568	4469

* N50 - sequence length of the shortest contig at 50% of the total genome length; L50- number of contigs length making up half of the genome size.

### 2.2 Whole genome sequencing

**Fig. 3:**
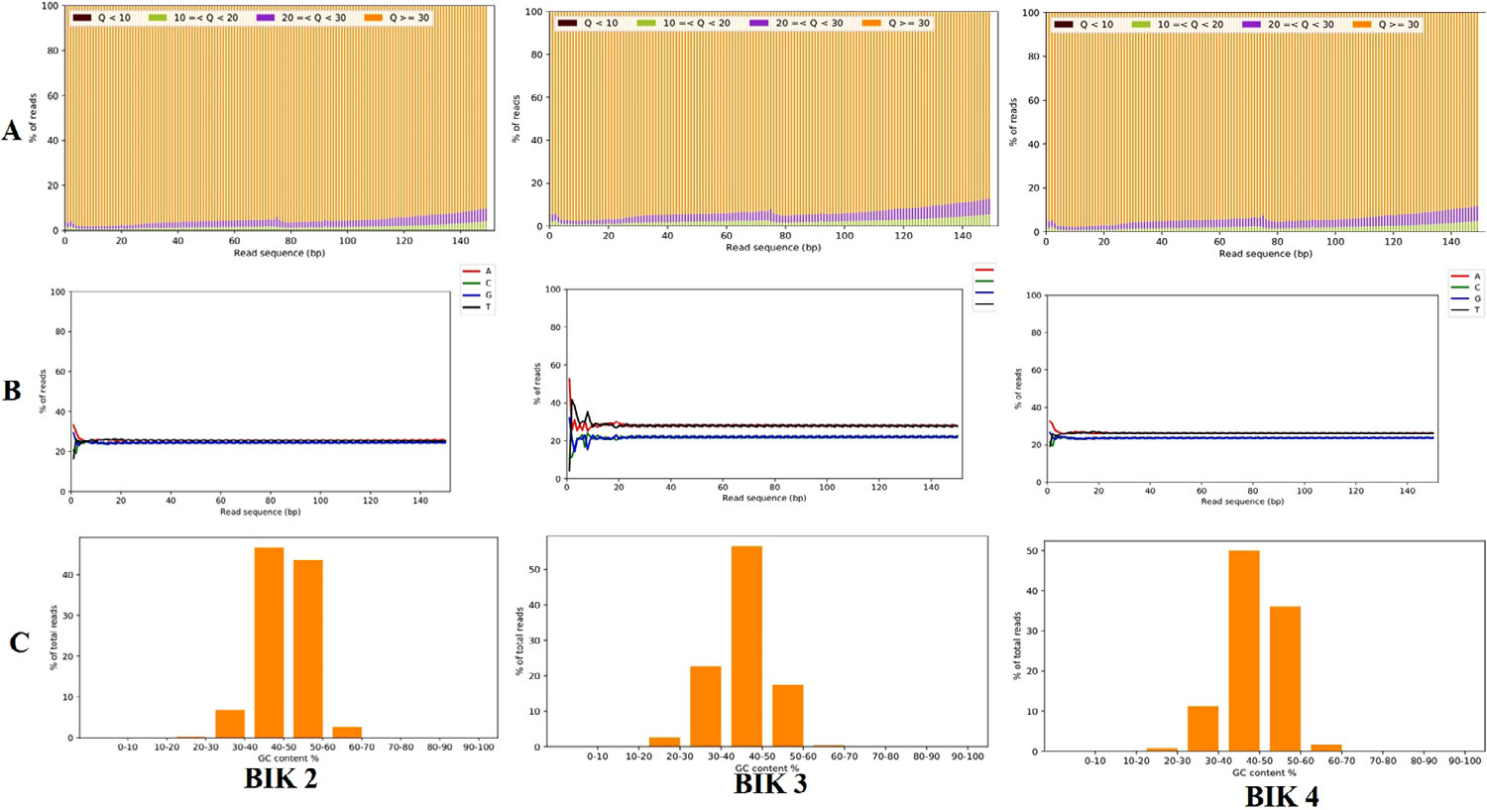
Quality check of three *Bacillus* strains (A) Quality distribution (B) Base distribution (C) GC distribution.

Whole Genome Sequencing (WGS) of three *Bacillus* isolates resulted in 20, 274, 842; 12, 674, 497 and 17, 571, 991 raw reads for BIK2, BIK3 and BIK4 respectively. The quality of raw sequence reads were assessed using Fast QC and then pre-processed using AdapterRemovalV2 version 2.3.1 tool [7] (Fig. 3) generating 20,260,548; 12,667,151 and 17,551,922 clean reads for BIK2, BIK3 and BIK4 with an average read length of 150 bp respectively, representing coverage of 1447X, 905X and 585X folds. The cleaned reads were *de novo* assembled using the Unicycler ver. 0.4.8 assembler [8] and CDSs in the assembled contigs were predicted using prodigal version 2.6.3 [10]. Completeness of the genome assembly was assessed by BUSCO ver. 4.0.6 [9] and quality of the genome assembly was assessed by QUAST ver. 4.6 [10]. Protein encoding genes were predicted using Prodigal ver. 2.6.3 [11].

**Fig. 4:**
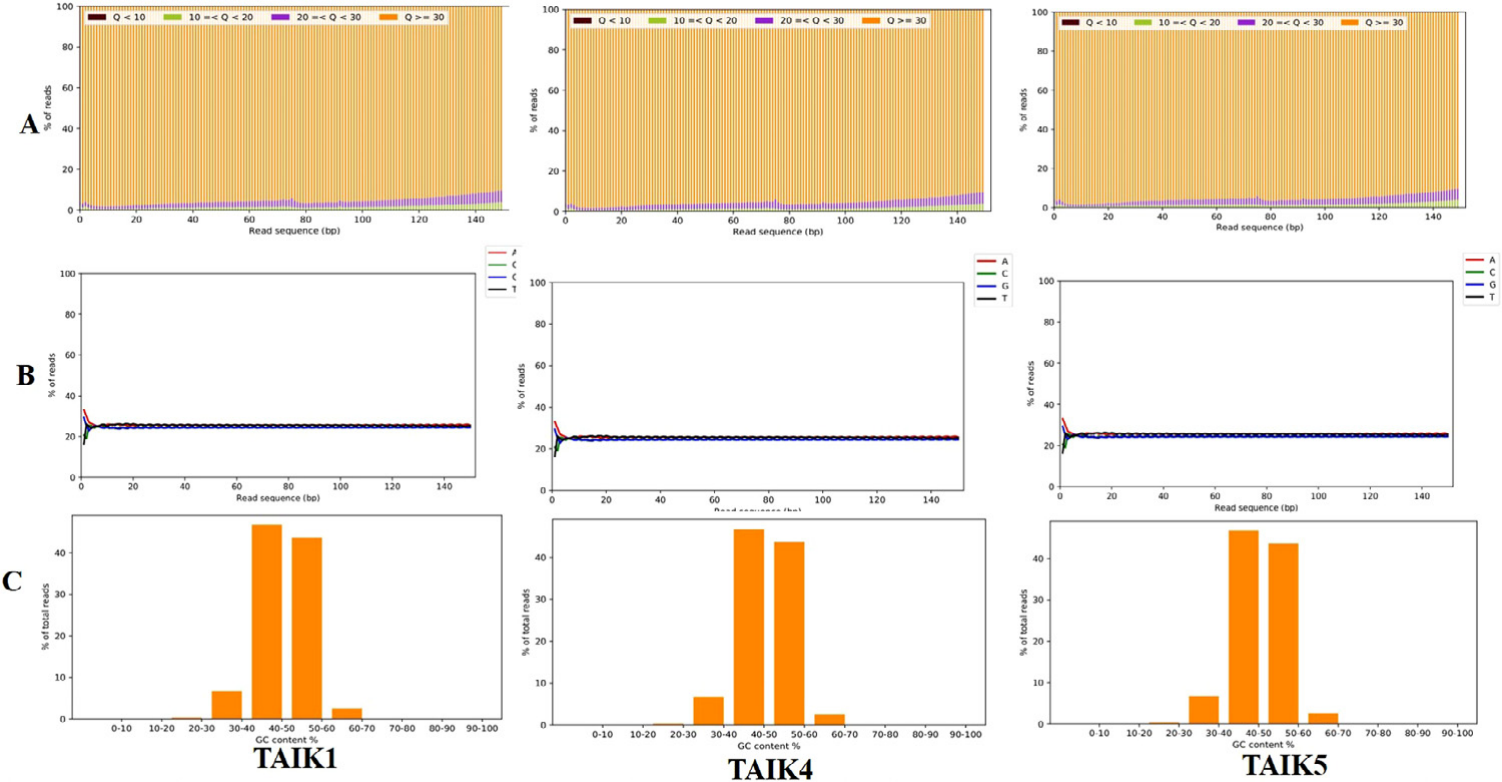
Quality check of three *Trichoderma* strains (A) Quality distribution (B) Base distribution (C) GC distribution.

For the *Trichoderma* strains TAIK1, TAIK4 and TAIK5, a total of 15, 230, 394; 16, 467, 915 and 20, 615, 262 raw reads were generated and the quality of these raw sequence reads were assessed using Fast QC and then pre-processed using AdapterRemovalV2 version 2.3.1 tool [7] (Fig. 4) resulting in 11,502,933; 14,374,041; 18,498,253 clean reads respectively with an average read length of 150 bp, representing coverage of 109X, 150X and 116X folds. *De novo* assembly was performed using the Velvet assembler version 1.2.10 (https://angus.readthedocs.io/en/2016/week3/LN_assembly.html) and CDSs in the assembled contigs were predicted using Augustus assembler version 3.4.0 (http://bioinf.uni-greifswald.de/augustus/). Completeness of the genome assembly was assessed by BUSCO ver. 4.0.6 [8] and quality of the genome assembly was assessed by QUAST ver. 4.6 [10]. Protein encoding genes were predicted using Prodigal ver. 2.6.3 [11]. Organism annotation was determined from the predicted genes which were compared with the Uniprot database using BlastX version 2.6.0 (ftp://ftp.ncbi.nlm.nih.gov/blast/executables/blast+/) program with E-value cut offset to 10^−3^ and subsequent filtering was done for the best hits based on the query coverage, identity and similarity score.

### 2.2 Assembly statistics

*Bacillus* isolates *viz.,* BIK2, BIK3 and BIK4 and *Trichoderma* isolates *viz.,* TAIK1, TAIK4 and TAIK5 consisted of 26, 28 and 30 contigs; 702, 473 and 449 with a maximum size of 1078,503; 575,880 and 1056,15 bp; 1048,585; 624,435 and 725,734 respectively. The sequence length of the shortest contig (N50) of *Bacillus* isolates *viz.,* BIK2, BIK3 and BIK4 and *Trichoderma* isolates *viz.,* TAIK1, TAIK4 and TAIK5 were 1029,777; 320,958 and 627,466; 226,906 and 161,701 respectively. While the length of the contig (L50) were two, five and three; 50, 64 and 70 for *Bacillus* and *Trichoderma* strains respectively. The sequencing data were deposited in the Sequence Read Archive (SRA) with accession numbers JAHWRC01, JAHKKH01 and JAHWRD01 for *Bacillus* strains BIK2, BIK3 and BIK4 respectively and JA1AZZ01, JA1CDU01 and JAHYXG01 accessions for *Trichoderma* strains TAIK1, TAIK4 and TAIK5 respectively. The closest associated strains to the isolates include *Bacillus velenzensis* (DSM23117) for BIK2, *Bacillus paralicheniformis* (ATCC 9945a) for BIK4 and *Trichoderma asperellum* (CBS443.97) for all the three *Trichoderma* strains *viz.*, TAIK1, TAIK4 and TAIK5. The Bio-project accession numbers are presented in the specifications table.

## Funding Information

This work was supported by ICAR- Indian Institute of Rice Research, Hyderabad, India.

## Ethical Statement

Not applicable.

## CRediT authorship contribution statement

**C. Kannan:** Conceptualization, Supervision, Writing – review & editing. **M. Divya:** Methodology, Writing – review & editing. **G. Rekha:** Methodology, Formal analysis, Writing – review & editing. **Kalyani M. Barbadikar:** Validation, Investigation, Writing – review & editing. **P. Maruthi:** Methodology. **S.K. Hajira:** Methodology, Project administration. **R.M. Sundaram:** Supervision.

## Declaration of Competing Interest

The authors declare that they have no known competing financial interests or personal relationships which have or could be perceived to have influenced the work reported in this article.

## Appendix Supplementary materials

Image, image 1

Image, image 2
