# Supplementary figures and images for: Whole genome sequencing data of native isolates of Bacillus and Trichoderma having potential biocontrol and plant growth promotion activities in rice
Source: Data Brief. 2022 Feb 4;41:107923. doi: 10.1016/j.dib.2022.107923 (PMC8847797; doi:10.1016/j.dib.2022.107923)

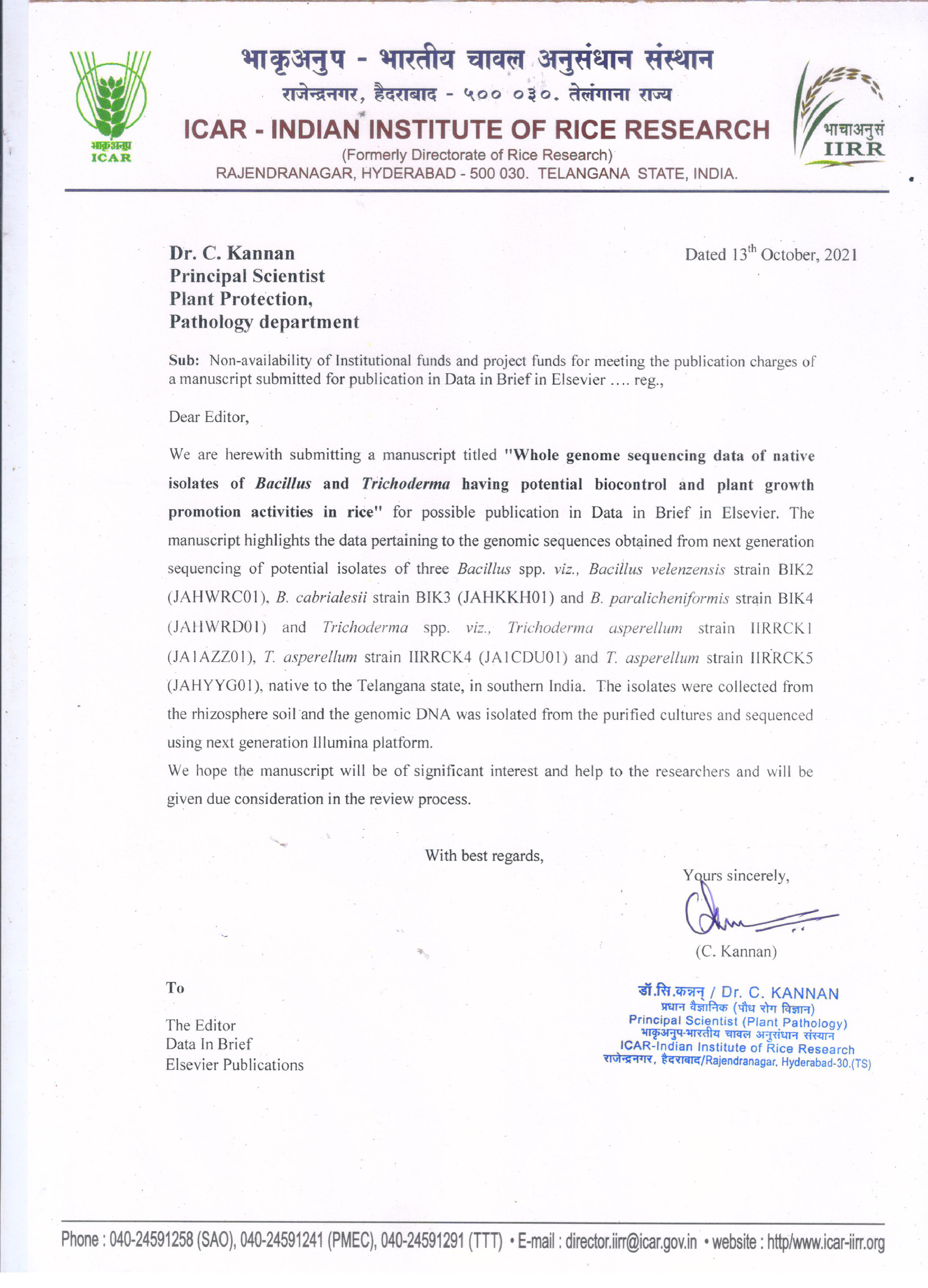

Supplement: Supplementary file 1 [file mmc1.jpg]

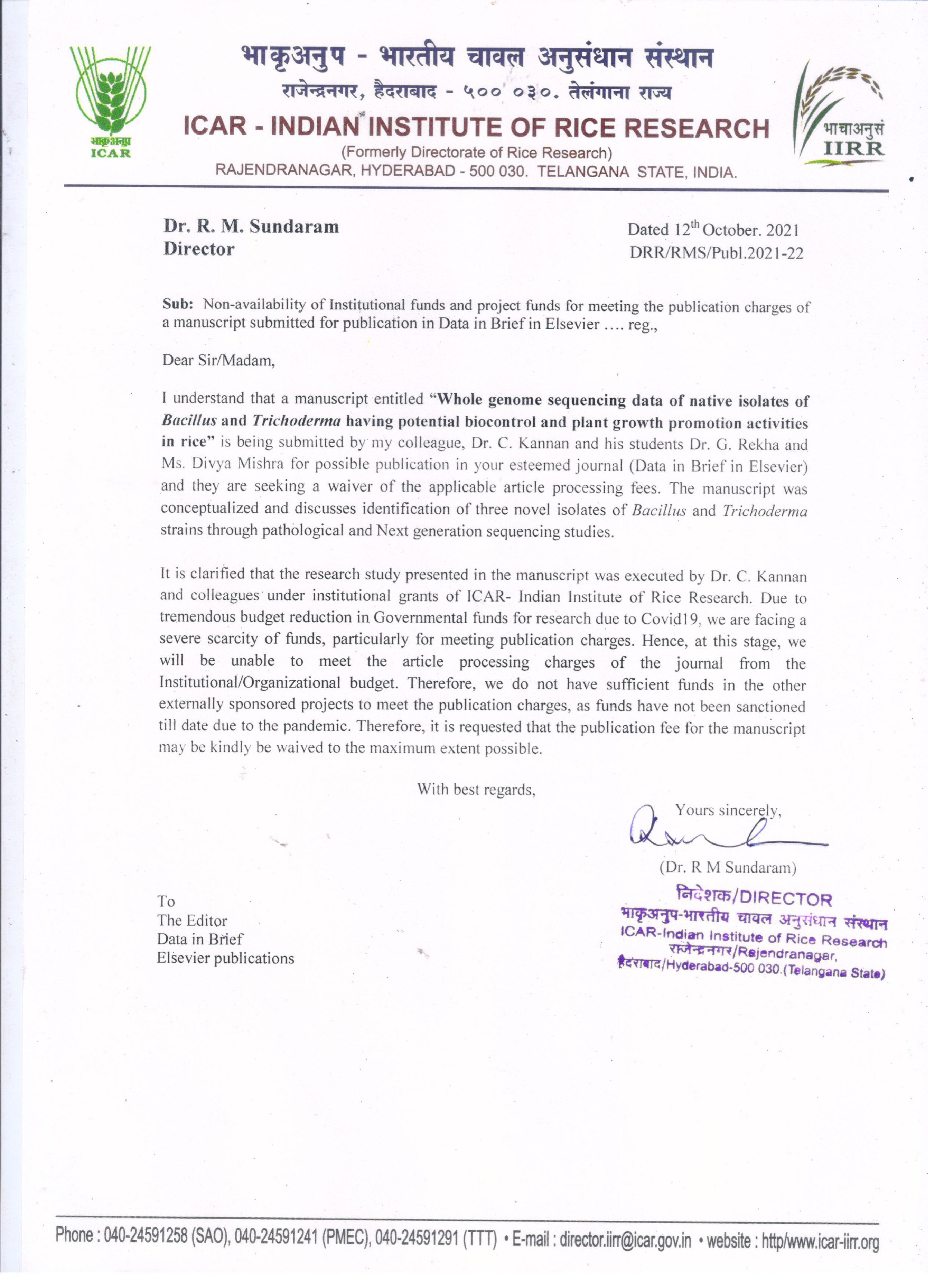

Supplement: Supplementary file 2 [file mmc2.jpg]
